# Trait rejection sensitivity is associated with vigilance and defensive response rather than detection of social rejection cues

**DOI:** 10.3389/fpsyg.2015.01516

**Published:** 2015-10-02

**Authors:** Taishi Kawamoto, Hiroshi Nittono, Mitsuhiro Ura

**Affiliations:** ^1^Japan Society for the Promotion of ScienceTokyo, Japan; ^2^Graduate School of Arts and Sciences, The University of TokyoMeguro-ku, Japan; ^3^Graduate School of Integrated Arts and Sciences, Hiroshima UniversityHigashi-hiroshima, Japan; ^4^Department of Psychology, Otemon Gakuin UniversityIbaraki, Japan

**Keywords:** social rejection, social inclusion, rejection sensitivity, evolutionary perspective, event-related brain potentials, P1, N170, late positive potential

## Abstract

Prior studies suggest that psychological difficulties arise from higher trait Rejection Sensitivity (RS)—heightened vigilance and differential detection of social rejection cues and defensive response to. On the other hand, from an evolutionary perspective, rapid and efficient detection of social rejection cues can be considered beneficial. We conducted a survey and an electrophysiological experiment to reconcile this seeming contradiction. We compared the effects of RS and Rejection Detection Capability (RDC) on perceived interpersonal experiences (Study 1) and on neurocognitive processes in response to cues of social rejection (disgusted faces; Study 2). We found that RS and RDC were not significantly related, although RS was positively related to perceived social rejection experiences and RDC was positively related to perceived social inclusion experiences. Event-related brain potentials (ERPs) revealed that higher RS was related to cognitive avoidance (i.e., P1) and heightened motivated attention (i.e., late positive potential: LPP), but not to facial expression encoding (i.e., N170) toward disgusted faces. On the other hand, higher RDC was related to heightened N170 amplitude, but not to P1 and LPP amplitudes. These findings imply that sensitivity to rejection is apparently distinct from the ability to detect social rejection cues and instead reflects intense vigilance and defensive response to those cues. We discussed an alternative explanation of the relationship between RS and RDC from a signal detection perspective.

## Introduction

People are sensitive to social rejection, because social glue is critical for us (Baumeister and Leary, 1995). Social connection with others is considered to have evolutionary benefit because it aids survival and reproduction (Williams, 2009; Wesselmann et al., 2013). In modern life, social rejection still affects our psychological adaptation in various ways, by increasing depression, aggression, and mortality (e.g., Leary et al., 2003; Nolan et al., 2003; Holt-Lunstad et al., 2010; Van Orden and Joiner, 2013). Thus people sensitively perceive and respond to social rejection. But what does “sensitivity” to social rejection exactly mean?

At least two lines of research have investigated sensitivity to social rejection, a trait perspective and an evolutionary perspective. Trait rejection sensitivity (RS) is defined as anxious expectation and ready perception of social rejection, and overreaction to it (Downey and Feldman, 1996). According to RS theory (Romero-Canyas et al., 2010), higher trait RS results in multiple psychological difficulties, including depression, aggression, and relational breakup (e.g., Downey and Feldman, 1996; Downey et al., 1998a,b, 2000; Ayduk et al., 1999, 2001; Marston et al., 2010; Romero-Canyas et al., 2010). From an evolutionary perspective, as explicated in the stage of coping theory (Williams, 2009), sensitively detecting social rejection cues is considered to have benefits: avoiding further social rejection and better coping with social rejection allows the person to regain social connections (Wesselmann et al., 2013). Thus, there appear to be multiple aspects of sensitivity to social rejection.

Rejection sensitivity theorists argue that the “sensitivity” aspect of their theory refers to (a) a heightened awareness and vigilance to social rejection cues, (b) the ability to differentially detect those cues, and (c) an allergic defensive reaction to those cues (Romero-Canyas et al., 2010). The second argument overlaps in part with the detection capability claim of stage of coping theory (Williams, 2009); however, the two theories predict different consequences: higher trait RS predicts more psychological difficulties, whereas detection capability predicts better outcomes. Although the first and third arguments—heightened vigilance and defensive reaction to social rejection cues—have been supported by a wealth of evidence (e.g., Downey and Feldman, 1996; Downey et al., 1998a, 1999, 2004; Downey and Romero-Cayas, 2005; Berenson et al., 2009; Ehrlich et al., 2015), direct evidence of the relationship between trait RS and the ability to detect social rejection cues is still limited.

What remains unclear is whether trait RS reflects *the capability of detecting social rejection cues*. To clarify this and to help reconcile the theories, we attempted to distinguish between trait RS and individual differences in the capability of detecting cues that may be related to social rejection (trait rejection detection capability: RDC). We used two different approaches—a survey (Study 1) and an electrophysiological experiment (Study 2)—to understand the relationship between trait RS and the capability of detecting social rejection cues.

### Trait Rejection Sensitivity

Rejection sensitivity is word that has often been used in social rejection literature, whereas the RS Scale has been commonly used as a trait measure (e.g., Downey and Feldman, 1996). Numerous studies have demonstrated that RS is related to psychological difficulties and other adverse outcomes: relationship breakup (e.g., Downey et al., 1998a), interpersonal aggression (e.g., Downey et al., 1998b, 2000; Romero-Canyas et al., 2010), and depression (e.g., Ayduk et al., 2001; Marston et al., 2010). These consequences stem mainly from heightened defensive motivational response and heightened vigilance, including poor emotional regulation capacities, in those high in RS (e.g., Downey et al., 2004; Downey and Romero-Cayas, 2005; Kross et al., 2007; Berenson et al., 2009; Romero-Canyas et al., 2010). People with high RS show heightened startle reflex response and attentional avoidance in response to threat cues related to social rejection (Downey et al., 2004; Berenson et al., 2009).

Why do people with high RS show defensive responses to social rejection cues? RS is assumed to develop in the context of early social rejection experiences and the lack of adequate relationships with others (Feldman and Downey, 1994; Downey et al., 1997). Such early experiences might lead to a tendency to generate anxious expectations of rejection, leading individuals to behave in a defensive manner. Other influential theories also suggest a relationship between chronic social rejection experiences and defensive motivation when responding to cues of social rejection. For example, the Stage of coping theory (Williams, 2009) suggests that long-term ostracism could lead people to a resignation stage, which results in avoidance and withdrawal behaviors. Optimal calibration theory (Chester et al., 2012) also emphasizes the important role of chronic social rejection experiences on the processing of social rejection stimuli. Using a life history framework, Chester et al. (2012) proposed that chronic social rejection experiences in early life could shift neural processing of social rejection to be avoidant and defensive. Therefore, the relationship between RS and defensive responses is supported by a wealth of evidence and theoretical frameworks.

There is evidence that people with high RS have enhanced ability to differentially detect to and heightened vigilance to social rejection cues. For example, people with high RS felt more distress in response to ambiguous social rejection (Downey and Feldman, 1996; Downey et al., 1998b) and reported higher conflictual ratings in response to their partners (Norona et al., 2014). Psychological responses to social threat cues also provide evidence regarding the differential detection, and heightened vigilance for social rejection cues. For example, people with high RS show a resistance to extinction of conditioned responses to threating faces, which is not observed for neutral faces, or to non-social stimuli (Olsson et al., 2013). Startle responses are also pronounced in high RS individuals, when responding to rejection-related paintings, but not to other negative or positive paintings (Downey et al., 2004). Although emotional and conflictual ratings offer valuable information, such studies do not demonstrate that the relationship between trait RS and detection capability is attributable to social rejection alone: people with low RS might have similar detection capability, but stronger emotional regulation skills may enable them to better regulate rejection-related feelings. In fact, there is evidence that people with low RS have better emotional regulation capacities (Kross et al., 2007). Moreover, previous studies have failed to demonstrate that the detection and vigilance for social rejection cues are explicitly dissociated. Either detection or vigilance can be interpreted from intense emotional and defensive responses. However, the detection and vigilance for social rejection cues are different processes. Detection of social rejection cues is a transient event that is characterized by the experience of a discrepancy, which sometimes evokes pain and anxiety (Eisenberger and Lieberman, 2004; Jonas et al., 2014). Vigilance, on the other hand, is a prolonged state that is characterized by the ease of attending and the sustained attention to social rejection cues, which disrupt attention to other features of the environment (Romero-Canyas et al., 2010). Therefore, the detection and vigilance for social rejection cues should be represented by different neural correlates, and be affected by different individual dispositions. Overall, the relationship between RS and the ability to detect social rejection cues requires further investigation.

### Sensitivity to Social Rejection from an Evolutionary Perspective

Social rejection decreases survival rates in mammals generally (Kling et al., 1970; Silk et al., 2003) and in humans specifically (Holt-Lunstad et al., 2010). Thus, from an evolutionary perspective, detection of cues indicating possible social rejection would seem to be vital for survival and reproduction (Williams, 2009; Wesselmann et al., 2013). Indeed, people are able to detect even the slightest hint of social rejection, which typically evokes aversive feelings (e.g., Williams et al., 2000; Zadro et al., 2004; van Beest and Williams, 2006; Gonsalkorale and Williams, 2007; Wirth et al., 2010; van Beest et al., 2011).

There is, however, less direct evidence linking the ability to detect social rejection cues and adaptive cognitive or behavioral tendencies, such as enhanced social inclusion experiences and/or improved relational functioning. There are at least three reasons for this. First, RS may reflect the anxious expectation of social rejection, which leads to heightened vigilance and defensive behavioral tendencies, rather than the capability of detecting social rejection cues *per se*. In other words, an individual could readily detect social rejection cues but not necessarily show the anxious anticipation that leads to heightened vigilance and defensive behaviors in response to those cues. Second, studies from an evolutionary perspective have focused mainly on the subtlest situations in which people were able to detect being rejected (e.g., Williams, 2009). Although these studies could be explained in terms of evolutionary benefit, more direct evidence is needed to strengthen the evolutionary argument (Wesselmann et al., 2013). Third, studies from both perspectives used emotional responses or need threats—belonging, self-esteem, control, and meaningful existence—to investigate sensitivity to social rejection (e.g., Downey and Feldman, 1996; Downey et al., 1998b; Williams et al., 2000; Zadro et al., 2004; Gonsalkorale and Williams, 2007; Williams, 2009). Although detection of social rejection is often accompanied by painful feelings and need threats (e.g., Williams et al., 2000; Eisenberger et al., 2003), detection of social rejection cues *per se* also involves perceptual and cognitive responses. People need to detect social rejection cues to change their behaviors to regain social inclusion and to avoid further rejection, but they may not have to explicitly feel negative emotions or need threats at all times. In fact, the stage of coping theory proposes that there is detection phase before emotional and threat responses to social rejection (Williams, 2009). To help bridge these gaps and directly investigate the relationship between RS and detection sensitivity to social rejection, we developed the RDC Scale.

### Trait Rejection Detection Capability

To measure RDC, we applied the idea of the *neural alarm system* proposed by Eisenberger and Lieberman (2004). They argued that two systems are needed for adequate operation of an alarm system: the first is a discrepancy monitoring system, which serves to detect deviations from desired standards, and the second is a sounding mechanism that signals a problem that needs to be addressed. We consider the discrepancy detection function to be associated with the detection of social rejection and social pain to be the product of the sounding system. In concurrence with these suggestions, previous findings suggest that the dorsal anterior cingulate cortex (dACC) underlies both functions (e.g., Eisenberger et al., 2003, 2011; Onoda et al., 2010; Kawamoto et al., 2012, 2015; Eisenberger, 2015; Rotge et al., 2015).

A discrepancy detection function concurs with the evolutionary perspective, which suggests that people should readily detect any kind of threatening cues (e.g., Zadro et al., 2004; Gonsalkorale and Williams, 2007; Williams, 2009; Wirth et al., 2010). Detecting potential cues of social rejection is more beneficial than missing the cues completely (Williams, 2009). Further, the stage of coping theory argues that dACC plays a key role in detection of social rejection cues. Thus, we focused on this discrepancy-detecting function, with the RDC Scale measuring the extent to which an individual notices a discrepancy in situations that may be related to social rejection. We did not directly ask about feelings of social rejection because such feelings are often accompanied by other negative emotions and distress (e.g., Williams et al., 2000; Eisenberger et al., 2003). Further, a discrepancy detection function of the neural alarm system would be more likely to involve perceptual and cognitive processing than emotional responses. In addition, stage of coping theory assumes that “detection” involves unelaborated processes, which are sometimes not done deliberatively and thoughtfully (Williams, 2009). Thus, measuring “the extent to which an individual notices a discrepancy in situations that may be related to social rejection” is more suitable for measuring detection capability of social rejection cues than direct assessment of rejection-related affect, as conceptualized by the neural alarm system model and stage of coping theory.

More broadly, the differences between RS and RDC could be explained by generalized threats theory (Jonas et al., 2014). According to this theory, all theoretical threats cause defensive states that are related to the behavioral inhibition system (BIS), including heightened vigilance and avoidance responses. This BIS state is considered to be muted by engaging in approach-oriented reactions and to be modulated by the dispositional behavioral activation system (BAS). The BIS and BAS states concur with differences between RS and RDC: RS is related to heightened vigilance and avoidance responses (Romero-Canyas et al., 2010), whereas RDC might have certain benefits that lead to social inclusion experiences by effectively coping with social rejection (Williams, 2009). Thus, RS might be characterized by heightened BIS related states and responses, whereas RDC might be associated with those of BAS.

Overall, the RDC Scale measures the capability to detect social rejection cues *per se*, rather than the anxious expectation of those cues, or RS. We thus anticipated that RDC and RS would be not significantly related, and people with high RDC would show evidence of interpersonal and cognitive functioning that is related to evolutionary benefit. More specifically, we predicted that people with high RDC would report more perceived social inclusion experiences (Williams, 2009; Wesselmann et al., 2013), and those with higher RS would report more perceived social rejection experiences (e.g., Feldman and Downey, 1994; Downey et al., 1997). In addition, we predicted that people with high RDC would show heightened cognitive processes related to accurate recognition of social rejection cues; people with high RS would show heightened vigilance and defensive cognitive responses to those cues (Romero-Canyas et al., 2010).

### Overview of Present Investigation

To investigate the relationship between trait RS and the capability of detecting social rejection cues, we conducted two studies using different methods: a survey (Study 1) and an electrophysiological experiment (Study 2). Using the survey, we investigated how RS and RDC relate to perception of interpersonal relationships, specifically, social rejection and social inclusion. We also recorded event-related brain potentials (ERPs) in response to a cue of social rejection, a disgusted face (e.g., Rozin et al., 1994; DeWall et al., 2009a; Kawamoto et al., 2014). We sought to understand the neurocognitive bases of RS by focusing on ERP components that reflect detection capability (N170) as well as defensive responses (P1) and heightened vigilance (late positive potential: LPP).

## Study 1

We assessed nostalgia, depression, social inclusion, and social rejection experiences as potential correlates of RS and RDC. To provide converging evidence about the relationship between RDC and social inclusion experiences, we assess perceived social inclusion experience as well as nostalgia, “a sentimental longing for the past” (Wildschut et al., 2006, p. 976). Nostalgia has been associated with a large array of positive psychological consequences (Sedikides et al., 2008). In particular, nostalgia bolsters social connectedness (Wildschut et al., 2006; Zhou et al., 2008) in that it seems to represent a repository of social connections (Wildschut et al., 2010). We measured nostalgia as a proxy variable for past experiences (e.g., in elementary and secondary schools) of social connectedness and inclusion. We also assessed depression as a measure of health status, and perceived social rejection and inclusion that people experienced during the past 3 months.

If the capability of detecting social rejection cues is critical for survival and reproduction (Williams, 2009), this sensitivity should be related to social inclusion experiences (Wesselmann et al., 2013). We therefore predicted that RDC would be positively related to both nostalgia and perceived social inclusion experiences. In addition, we predicted that RS would be positively related to both depression and perceived social rejection experiences, as has been found in previous studies (e.g., Feldman and Downey, 1994; Downey et al., 1998a; Marston et al., 2010).

### Method

#### Participants

The original sample consisted of 184 university students (92 females) who were recruited from an introductory psychology class. The final sample consisted of 116 students (56 females, *M_age_* = 18.5, *SD* = 0.70) who participated at both assessment points. All participants gave written informed consent and took part in exchange for partial course credit. The Research Ethics Committee of the Graduate School of Integrated Arts and Sciences of Hiroshima University approved the study protocol.

#### Measures

##### Social Rejection Detection Capability Scale

The RDC Scale contains 13 items, which are adapted from a social exclusion experience questionnaire (Masui et al., 2013). Participants were told that people occasionally notice discrepancies in situations, because the situation is unwanted, or relatively unusual. Participants were then required to rate the degree of discrepancy they noticed in the thirteen situations on a 9-point scale ranging from 1 (*Not at all)* to 9 (*Very much*). The included items such as, *When you ask your friends to attend a class with you, they refuse* (Cronbach α = 0.91 at Time 1, Cronbach α = 0.93 at Time 2). See the appendix for the complete RDC Scale.

##### Rejection sensitivity questionnaire (RSQ)

The RSQ assesses individuals’ anxious expectations regarding rejection (Downey and Feldman, 1996). We used a Japanese version of the RSQ (Honda and Sakurai, 2000; Kawamoto and Ura, 2011) that includes the same items as the original RSQ, except for some minor changes (e.g., *dance party* was changed to *dinner party*). The measure consists of a series of situations in which rejection by a friend or significant other is possible (e.g., *You ask your friend to do you a big favor*). For each situation, participants rated the level of anxiety or concern that they would experience about the outcome of the situation using a 6-point scale ranging from 1 (*Very unconcerned*) to 6 (*Very concerned*) and the likelihood that the person with whom they are interacting in the situation would respond in an accepting manner also using a 6-point scale ranging from 1 (*Very unlikely)* to 6 (*Very likely*). Because the measure seeks to capture anxious expectations of rejection, a score for each situation is computed by weighing the likelihood of rejection according to the level of anxiety about the situation. To this end, the expected acceptance rating was reverse coded to indicate the expectation of rejection and was then multiplied by the degree of anxiety experienced in the situation. A cross-situational, total RSQ score was computed by obtaining a mean score across the situations described in the questionnaire (Cronbach α = 0.84).

##### Nostalgia

The nostalgia assessment used here was adapted from a previous study (Routledge et al., 2008). We measured this experience using three items that assess sentimental longing for the past (e.g., *How often do you look nostalgically back on your time in elementary and secondary schools?*). Participants provided ratings on a 9-point scale ranging from 1 (*Not at all*) to 9 (*Very often*: Cronbach α = 0.69).

##### Depression

Depression was measured using 10 items that comprise part of the Todai Health and Personality Inventory (Aoki et al., 1974). Participants rated their feelings on a 3-point scale that included 1 (*No*), 2 (*Neither Yes nor No*) and 3 (*Yes*). The scale has high internal consistency (Cronbach α = 0.86).

##### Social rejection and inclusion experiences

Social rejection and inclusion experiences were measured via a social exclusion experience questionnaire used in a previous study (Masui et al., 2013). This scale contains 11 items that measure social rejection experiences (e.g., *When I asked my friends if I could borrow their things, they said no*) and six items that measure social inclusion experiences (e.g., *My friends asked me to go to shopping*). Participants rated how often they experienced the scenarios during the past 3 months, from 0 (*Not at all*) to 4 (*Very often*). Both components have high internal consistencies (social rejection: Cronbach α = 0.86; social inclusion: Cronbach α = 0.84).

#### Procedure

The present study included two assessment points. Participants were asked to complete the RDC, RSQ, nostalgia, and depression scales at the first assessment point (Time 1: April). At the second assessment point (Time 2: July) approximately 3 months later, they were asked to complete the RDC to confirm test-retest reliability, and to complete the social exclusion experience scales to assesses interpersonal experiences during the first semester at a university in Japan (i.e., from April to July). All scales were counterbalanced.

### Results and Discussion

We conducted an exploratory principal axis factor analysis with promax rotation on responses to the RDC Scale. We conceptually expected that the measure of RDC would have a one-factor structure, because people need to detect all kinds of social rejection cues (e.g., Williams, 2009). However, the analysis indicated three factors with eigenvalues greater than 1. Therefore, we have first reported the results of total RDC scores, and then the results of the three RDC subcomponents to examine meaningful differences between the subcomponents.

The first factor accounted for 49% of the variance, compared with only 12 and 9% for the second and third factors, respectively. All items loaded greater than 0.30 on the first factor, and correlated above 0.40 with the corrected item total. The RDC was confirmed to have high internal reliability (Cronbach α = 0.91) and test-retest reliability (*r* = 0.72, *p* < 0.001). The test–retest reliability of the RDC scale was similar to that of the RS scale (*r* = 0.78; Downey and Feldman, 1996).

**Table 1** shows the mean values, standard deviations, and zero-order correlation coefficients among all variables with regard to the results of total RDC scores. Importantly, RDC scores were not significantly related to RS scores. As we predicted, RDC and RS were differentially related to nostalgia, depression, social rejection experiences, and social inclusion experiences. RDC was positively related to nostalgia and social inclusion experiences and negatively related to social rejection experiences. These relationships were still significant or marginally significant after controlling for RS (*r* = 0.28, *p* = 0.002 for nostalgia; *r* = 0.22, *p* = 0.018 for social inclusion experiences; *r* = -0.17, *p* = 0.063 for social rejection experiences). On the other hand, RS was negatively related to nostalgia and social inclusion experiences and positively related to social rejection experiences. These relationships were still significant or marginally significant after controlling for RDC (*r* = 0.19, *p* = 0.043 for social rejection experiences; *r* = -0.29, *p* = 0.002 for nostalgia; *r* = -0.18, *p* = 0.060 for social inclusion experiences). We also conducted Poisson and negative binomial regression analyses on the total social rejection experiences scores by entering RS and RDC scores as predictor variables. Both analyses once again indicated that RS tended to be positively related to social rejection experiences (Poisson: *B* = 0.037, *p* = 0.079, negative binomial: *B* = 0.033, *p* = 0.083) whereas RDC was not significantly related (Poisson: *B* = -0.066, *p* = 0.172, negative binomial: *B* = -0.073, *p* = 0.160). Finally, RS was positively related to depression, whereas RDC showed no relationship with this mental health variable. RS and depression were still positively correlated after controlling for RDC (*r* = 0.40, *p* < 0.001).

**Table 1:** Means, standard deviations, and zero-order Pearson correlation coefficients with regard to RDC subcomponent scores.

	*M*	*SD*	2	3	4	5	6	7
RS (T1)	13.66	3.17	-0.06	-0.29^∗^	0.40^∗^	0.20^∗^	-0.19^∗^	-0.07
RDC (T1)	5.89	1.46	–	0.28^∗^	0.09	-0.18^∗^	0.23^∗^	0.72^∗^
Nostalgia (T1)	3.72	0.87	–	–	-0.15	-0.03	0.25^∗^	0.17
Depression (T1)	1.66	0.48	–	–	–	0.32^∗^	-0.18	0.08
SR (T2)	0.73	0.52	–	–	–	–	-0.03	-0.15
SI (T2)	2.45	0.83	–	–	–	–	–	0.21^∗^
RDC (T2)	5.69	1.54	–	–	–	–	–	–

*T1, time 1; T2, time 2; RDC, rejection detection capability; RS, rejection sensitivity; SR, social rejection experiences; SI, social inclusion experiences; ^∗^p < 0.05*.

Three factors were retained as subcomponents of RDC (see appendix) by the scree test as noted before. The first factor included four items (e.g., Your friends all go to hang out somewhere but exclude you) in which participants are rejected without any direct sign of social rejection. We thus categorized the first factor as “indirect rejection” (Cronbach α = 0.92). The second factor included four items (e.g., When you send an e-mail to your friends, you get no replies), in which participants might feel they are rejected without any direct sign of social rejection. We thus categorized the second factor as “minimal rejection” (Cronbach α = 0.86). The third factor included five items in which there are direct signs of social rejection. We thus categorized the third factor as “direct rejection” (Cronbach α = 0.75). Subcomponents of these three factors were positively correlated with each other (*rs* > 0.35, *ps* < 0.001) and had relatively high test-retest reliabilities (indirect rejection: *r* = 0.65, *p* < 0.001, minimal rejection: *r* = 0.68, *p* < 0.001, direct rejection: *r* = 0.62, *p* < 0.001). We also conducted confirmatory factor analysis of RDC scores using RDC scores at Time 2. The three factor model (*x^2^* = 157.9, *df* = 62, *p* < 0.001, CFI = 0.919, RMSEA = 0.116, AIC = 426.0) fitted the data better than the single factor model (*x^2^* = 374.0, *df* = 65, *p* < 0.001, CFI = 0.739, RMSEA = 0.203, AIC = 215.9), Δ*x^2^* = 216.1, Δ*df* = 3, *p* < 0.001.

Importantly, none of the subcomponent scores were related to RS (see **Table 2**). In addition, indirect rejection and direct rejection scores showed the identical correlation pattern with those of RDC total scores. However, although minimal rejection scores were positively correlated with nostalgia, there were no significant relationships between minimal rejection and social inclusion/rejection scores.

**Table 2:** Means, standard deviations, and zero-order Pearson correlation coefficients with regard to RDC subcomponent scores.

	*M*	*SD*	RS (T1)	Nostalgia (T1)	Depression (T1)	SR (T2)	SI (T2)
RDC-IR (T1)	6.57	2.11	-0.09	0.28^∗^	-0.01	-0.23^∗^	0.27^∗^
RDC-MR (T1)	3.70	1.80	0.02	0.20^∗^	0.17	0.03	0.08
RDC-DR (T1)	7.09	1.33	-0.09	0.25^∗^	0.06	-0.26^∗^	0.23^∗^

*T1, time 1; T2, time 2; RDC, rejection detection capability; RS, rejection sensitivity; SR, social rejection experiences; SI, social inclusion experiences; IR, indirect rejection; MR, minimal rejection; DR, direct rejection; ^∗^p < 0.05*.

Study 1 demonstrated that RS and RDC were not related, and both traits were differentially related to perceived interpersonal relationships. Higher RS was related to more perceived social rejection experiences; higher RDC was related to more perceived social inclusion experiences. These findings provide initial evidence that RS is unrelated to the capability of detecting social rejection cues *per se*, and that RDC has some benefit in interpersonal functioning because it increases perception of social inclusion experiences. Unexpectedly, we also found some differences between each RDC subcomponent. We have discussed RDC subcomponents in the Section “General Discussion” below.

One limitation of Study 1 was that no direct evidence of the relationship between RDC and the capability for detecting rejection cues was observed. Therefore, in Study 2, we examined the relationship between RDC and the capability for detecting social rejection cues by using a more relevant task and applying electrophysiological methods.

## Study 2

To provide converging evidence, we conducted an ERP experiment to further investigate the relationship between RS and the capability of detecting social rejection cues. We focused on reactions to facial expressions, an approach that has been widely used in previous social rejection studies (Burklund et al., 2007; Bernstein et al., 2008, 2010; DeWall et al., 2009a). Disgusted faces present cues that indicate social threats and social rejection, whereas smiling faces present cues that suggest social inclusion (e.g., Rozin et al., 1994; Parkinson, 2005; DeWall et al., 2009a; Kawamoto et al., 2014).

Of the various ERP components, we focused *a priori* on P1, N170, and LPP, which have been indicated as indices of defensive responses, and indices of detection, and vigilance in response to emotional and facial stimuli. P1 is a lateral occipital positive ERP component that occurs at around 80–100 ms post stimulus onset and has been linked to visual attention (Hillyard and Anllo-Vento, 1998). A previous study revealed that reduced P1 amplitude in response to threatening faces reflects cognitive avoidance (Jetha et al., 2012). N170 is an occipito-temporal negative ERP component which occurs at around 170 ms. The amplitude of this component increases in response to faces as compared to non-face stimuli (e.g., Bentin et al., 1996; Johnston et al., 2014). N170 appears to reflect early facial perception or structural encoding of faces (e.g., Bentin et al., 1996; Eimer, 2000) and relates to emotional face recognition accuracy (e.g., Tamamiya and Hiraki, 2013). LPP is a positive ERP component that occurs about 400 ms after stimulus onset. LPP amplitude is larger for emotional faces than for neutral faces (e.g., Eimer et al., 2003; Eimer and Holmes, 2007). Generally, LPP amplitude reflects the extent of motivated attention and/or emotional regulation (e.g., Hajcak et al., 2010; Paul et al., 2013).

Based on previous studies (Downey et al., 2004; Kross et al., 2007; Berenson et al., 2009) and the results of Study 1, we formulated four hypotheses. First, RS would be negatively related to P1 amplitude in response to disgusted faces—people with high RS would show reduced P1 amplitude, due to cognitive avoidance (Downey et al., 2004; Berenson et al., 2009). Second, RS would be unrelated to N170 amplitude in response to disgusted faces because RS and RDC are unrelated. Third, RS would be positively related to LPP amplitude in response to these faces, such that people with high RS would show increased LPP amplitude because of the motivational salience of the stimuli and their relatively poor emotional regulation skills (Kross et al., 2007; Romero-Canyas et al., 2010). Fourth, RDC would be unrelated to P1 and LPP amplitudes but negatively related to N170 amplitude: People high in RDC would show greater N170 amplitude in response to disgusted faces because they more accurately detect and process rejection cues (Williams, 2009; Wesselmann et al., 2013). We tentatively explored relationships between subcomponents of RDC and ERP components without forming any specific hypothesis.

### Method

#### Participants

Thirty-five healthy undergraduate students (17 females; *M_age_* = 18.46, *SD* = 0.70) participated in the experiment. All were right-handed (Oldfield, 1971). The Research Ethics Committee of the Graduate School of Integrated Arts and Sciences of Hiroshima University approved the protocol. All participants gave written informed consent.

#### Procedure

After completing the Japanese version of the RSQ (Downey and Feldman, 1996; Kawamoto and Ura, 2011; Cronbach α = 0.85) and RDC Scale (Cronbach α = 0.93 for total scores, Cronbach α = 0.94 for indirect rejection scores, Cronbach α = 0.85 for minimal rejection scores, Cronbach α = 0.82 for direct rejection scores), participants performed a facial expression viewing task that was similar to tasks used in previous studies (e.g., Leppanen et al., 2007; Kawamoto et al., 2014).

#### Stimulus and Task

Event-related brain potentials were recorded while participants passively viewed color pictures of individual female and male models (two females and two males) with neutral, smiling, and disgusted facial expressions. The pictures were selected from the ATR facial expression database (DB99). Stimulus presentation was controlled by Inquisit 3.0 (Millisecond Software) running on a desktop computer, and the stimuli subtended approximately 12° × 16°, at a viewing distance of 60 cm. The presentation of each facial stimulus was preceded by presentation of a fixation cross at the center of the screen. After a randomly varying interval ranging from 1,500 to 2,500 ms, the fixation cross was replaced by a facial stimulus presented for 1,000 ms. Each facial expression was presented 52 times, for a total of 156 test trials. In addition, so that participants would maintain attention to the task, they were required to respond to a checkerboard pattern that was presented on 13 additional trials. The task therefore consisted of a total of 169 trials and lasted about 8 min.

#### ERP Recording and Processing

An electroencephalogram (EEG) was recorded at 39 scalp sites using Ag/AgCl electrodes on an elastic cap. Vertical and horizontal electrooculograms were recorded from electrodes attached above and below the left eye and at the outer canthi. Electrode impedances were less than 20 KΩ. The signal was recorded with a bandpass filter of 0.016–60 Hz, at a sampling rate of 1000 Hz and re-referenced to the nose tip. An FIR filter of 0.1–30 Hz was applied to the ERP components. Ocular artifacts were corrected using the method of Gratton et al. (1983), implemented in Brain Vision Analyzer 2.02 (Brain Products, Germany). ERP waveforms were obtained by averaging a 1200-ms period from 200 ms before to 1000 ms after the onset of a facial stimulus (smiling, neutral, or disgusted face). For each ERP component, we chose time windows and electrodes providing representative values based on previous studies (e.g., Rubin et al., 2011; Kawamoto et al., 2014), as well as on the topographical distribution of grand-averaged ERP activity. The mean amplitude of P1 was measured at O1 and O2 90–130 ms after stimulus onset. The mean amplitude of N170 was measured at T5 and T6 140–190 ms after stimulus onset. The mean amplitude of LPP was measured at Pz 400–600 ms after stimulus onset.

#### Data Analysis

To test the effects of emotional valance and laterality on ERP components, we conducted a 3 (facial expression: smile vs. neutral vs. disgust) × 2 (electrode: O1 vs. O2 for P1, T5 vs. T6 for N170) ANOVA on P1 and N170 component amplitudes. For the LPP component, we performed a one-way ANOVA (facial expression: smile vs. neutral vs. disgust). Significant results were examined using *post hoc* analyses. The Bonferroni procedure was used to correct multiple comparisons.

To test our hypotheses, we calculated Pearson correlation coefficients between the questionnaire scores (RS and RDC) and ERP responses (P1, N170, and LPP) to disgusted faces. The mean amplitude values of the left and right electrodes were used for this correlation analysis because the effect of electrode was not statistically significant, as described in the following section. We then conducted the same correlation analysis on responses to neutral and smiling faces to examine whether RS and RDC are only affected by disgusted faces. We also conducted the identical analysis described above with RDC subcomponents.

### Results and Discussion

**FIGURE 1:**
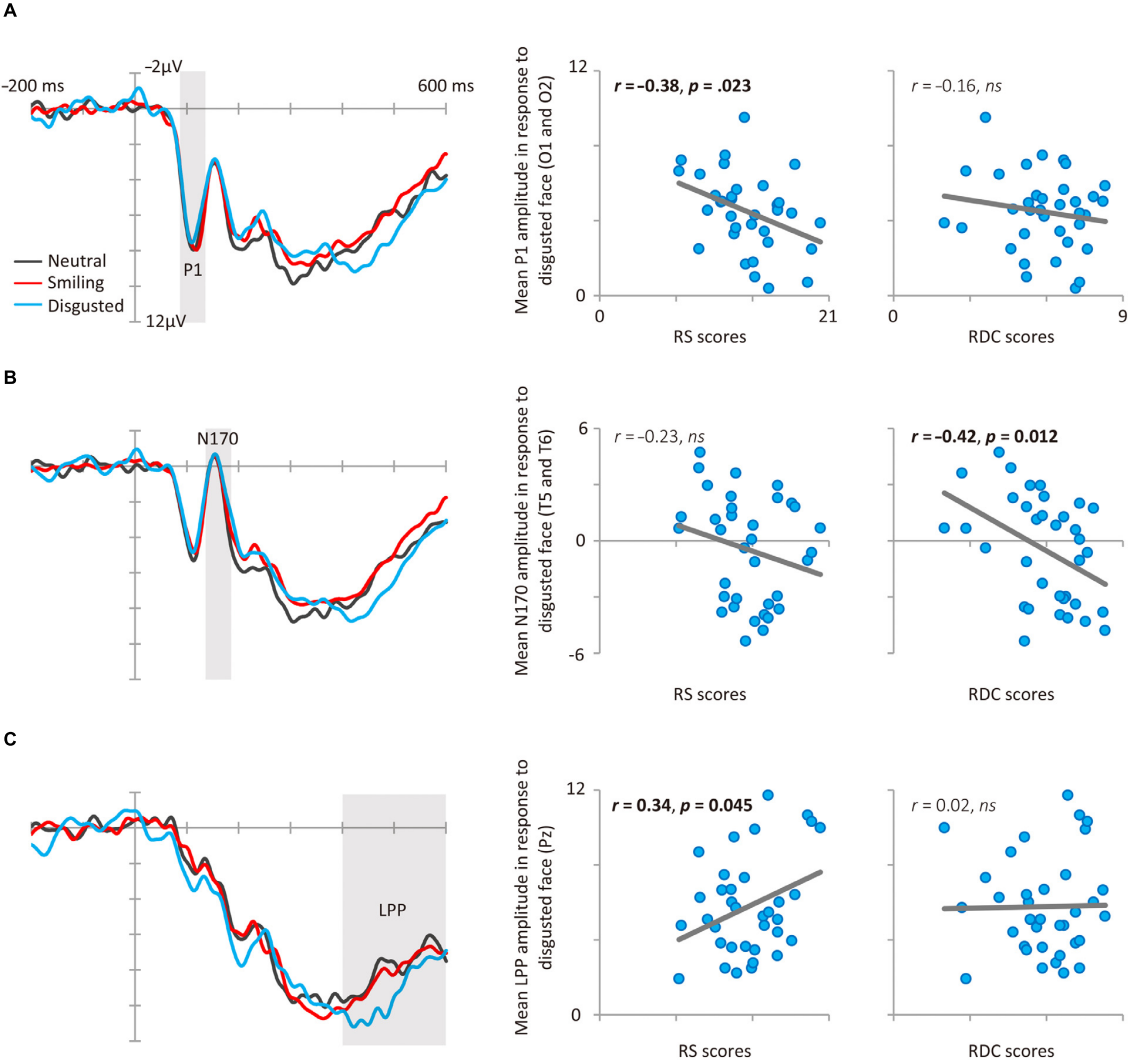
**Results of Study 2. (A)** P1 event-related brain potential (ERP) results. Average grand mean waveforms (O1 and O2) for facial expressions (left). Correlations between P1 amplitude in response to disgusted faces and RS (middle), RDC (right). **(B)** N170 ERP results. Average grand mean waveforms (T5 and T6) for facial expressions (left). Correlations between N170 amplitude in response to disgusted faces and RS (middle), RDC (right). **(C)** LPP ERP results. Grand mean waveforms for facial expressions at Pz (left). Correlations between LPP amplitude in response to disgusted faces and RS (middle), RDC (right).

**FIGURE 2:**
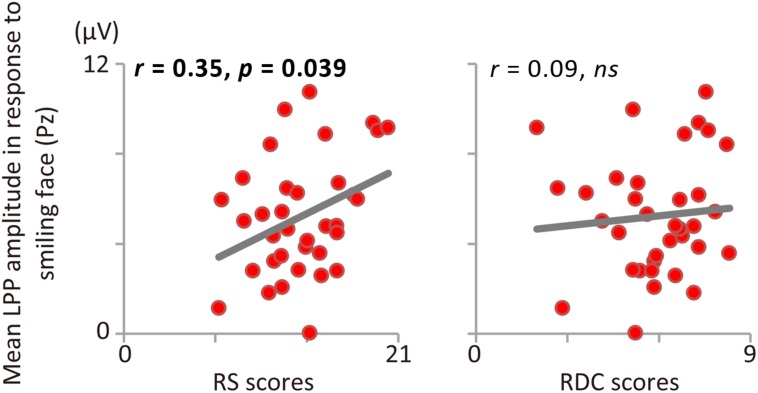
**Correlations between LPP amplitude in response to smiling faces and RS (left), RDC (right)**.

Consistent with Study 1, RS and RDC were unrelated (*r* = 0.13, *p* = 0.45). **Figure 1** shows the results of ERP and scatter plots of each trait score (RS and RDC total scores) and each ERP component (P1, N170, and LPP) in response to disgusted faces. **Figure 2** shows scatter plots of each trait score (RS and RDC total scores) and LPP amplitude in response to smiling faces. **Table 3** summarizes all correlation coefficients between trait scores and ERP components.

**Table 3:** Pearson correlation coefficients between trait measures and ERP components.

	P1			N170			LPP		
	Neutral	Smile	Disgust	Neutral	Smile	Disgust	Neutral	Smile	Disgust
RS	-0.08	-0.08	-0.38^∗^	-0.06	0.01	-0.23	0.12	0.35^∗^	0.34^∗^
RDC-total	-0.18	0.16	-0.16	-0.20	-0.23	-0.42^∗^	0.10	0.09	0.02
RDC-IR	-0.17	0.11	-0.12	-0.04	-0.17	-0.26	0.03	-0.05	-0.13
RDC-MR	-0.10	0.17	-0.11	-0.15	-0.11	-0.31	0.13	0.22	0.20
RDC-DR	-0.25	0.17	-0.17	-0.33	-0.38^∗^	-0.51^∗^	0.02	-0.01	-0.07

*RS, rejection sensitivity; RDC, rejection detection capability; IR, indirect rejection; MR, minimal rejection; DR, direct rejection, ^∗^p < 0.05*.

The facial expression (smile, neutral, and disgusted) × electrode (O1 and O2) ANOVA for P1 amplitude did not reveal any significant effects, *Fs* < 1.56, *ps* > 0.22, $\eta_{p}^{2}$ < 0.04. As we predicted, RS was negatively correlated with P1 amplitude in response to disgusted faces (see **Figure 1A**). This relationship was still significant after controlling for RDC (*r* = -0.37, *p* = 0.031). RDC was not significantly correlated with P1 amplitude in response to disgusted faces. P1 amplitudes in response to neutral and smiling faces were not significantly related to either RS or RDC scores.

The ANOVA for N170 amplitude revealed a marginally significant main effect of facial expression, *F*(2,68) = 2.60, *p* = 0.083, 𝜀 = 0.971, $\eta_{p}^{2}$ = 0.07, indicating that N170 amplitude was slightly larger for disgusted faces than for neutral faces (*p* = 0.064). Neither the main effect of electrode nor the interaction was significant, *F*s < 0.94, *p*s > 0.34, $\eta_{p}^{2}$ < 0.03. As we predicted, RDC was negatively correlated with N170 amplitude in response to disgusted faces (see **Figure 1B**). This relationship was still significant after controlling for RS (*r* = -0.41, *p* = 0.017). RS was not significantly correlated with N170 amplitude in response to disgusted faces. N170 amplitudes in response to neutral and smiling faces were not related to RS or RDC scores.

The ANOVA for LPP amplitude revealed a significant main effect of facial expression, *F*(2,68) = 5.99, *p* = 0.004, 𝜀 = 0.898, $\eta_{p}^{2}$ = 0.15, indicating that LPP amplitude was larger for disgusted than for neutral faces (*p* = 0.008). RS was positively correlated with LPP amplitude in response to disgusted faces (see **Figure 1C**). This relationship was still significant after controlling for RDC (*r* = 0.34, *p* = 0.048). In addition, LPP amplitude in response to smiling faces was positively correlated with RS (**Figure 2**). RDC was not significantly correlated with LPP amplitude in response to disgusted and smiling faces. Finally, LPP amplitude in response to neutral faces was not related to RS or RDC.

None of the RDC subcomponents were related to RS (*p*s > 0.52). All RDC subcomponents showed negative correlation with N170 amplitude in response to disgusted faces (see **Table 3**), however, only direct rejection scores were statistically significant (*p* = 0.130 for indirect rejection scores, *p* = 0.075 for minimal rejection scores). In addition, direct rejection scores were negatively related to N170 in response to smiling faces.

Study 2 provides further evidence about the relationship between RS and the capability of detecting social rejection cues. RS was related to cognitive avoidance (i.e., P1) and motivated attention (i.e., LPP), but not to facial encoding (i.e., N170) toward disgusted faces; RDC was related to increased N170 amplitude in response to disgusted faces, but not to P1 or LPP amplitudes. RS was again unrelated to the capability of detecting social rejection cues; instead, it reflects an intense defensive response and vigilance to those cues. In replicating and extending the results of Study 1, we used a more relevant task and demonstrated a relationship between RDC and the ability to detect rejection cues.

## General Discussion

People sensitively perceive and respond to social rejection. These sensitivities are in part modulated by trait RS, which encompasses multiple sensitivities to social rejection—heightened vigilance, detection capability, and defensive responses. By focusing on two dispositions—trait rejection sensitivity and trait RDC—and using two different methods—a survey (Study 1) and an electrophysiological experiment (Study 2), we sought to provide converging evidence about possible effects of trait RS on the ability to detect social rejection cues.

We found that RS and RDC were not significantly related. Using the survey method, we found that higher RS was related to the perception of more social rejection experiences, whereas higher RDC was related to the perception of more social inclusion experiences. Using the electrophysiological method, we revealed that these two dispositions also differ in their associations with cognitive processing of cues of social rejection, disgusted faces. RS was related to cognitive avoidance (as indexed by P1) and to enhanced motivated attention and/or poor emotional regulation (LPP); RDC was related to enhanced facial encoding processing (N170). We also found a positive correlation between RS scores and LPP amplitude in response to smiling faces, and three subcomponents of RDC.

These findings represent a first step in reconciling previous theories by showing that trait RS does not reflect the capability of detecting social rejection cues. RS theory (Romero-Canyas et al., 2010) proposed that people with high trait RS have heightened sensitivity with regard to vigilance, detection capability, and defensive response to social rejection cues, which definitely leads to psychological difficulties. On the other hand, stage of coping theory (Williams, 2009) claims that detection capability has an evolutionary benefit. Our findings support this argument from an evolutionary perspective and provide preliminary evidence that the ability to detect social rejection cues has some advantage: it leads to greater perception of social inclusion experiences. On the other hand, our findings partially support RS theory, in that trait RS reflects aberrant vigilance (LPP) and defensive responses (P1) to cues of social rejection; however, it does not appear to reflect detection capability (N170).

So how is trait RS in fact related to the capability of detecting social rejection cues? We propose that trait RS may influence interpretation bias rather than the ability to detect social rejection cues. There is some indirect evidence that supports our claim. For example, trait RS has been shown to be unrelated to accuracy in the discrimination of facial expressions (Pickett et al., 2004). In addition, people with low RS underestimate perceived negativity when evaluating self-relevant video clips that show someone reading the participant’s profile (Romero-Canyas and Downey, 2013). Thus, trait RS may affect bias in interpretation of cues of social rejection, but not the ability to detect them. Signal detection theory (e.g., Green and Swets, 1966; Lynn and Barrett, 2014) offers more clear-cut evidence for our proposal by providing two distinct perceptive sensitivity indexes: detection sensitivity and response bias. Detection sensitivity reflects the accuracy with which signal and noise are dissociated; response bias reflects the overall tendency to respond signal regardless of whether or not the stimuli are actual signals. Given our results and those of previous studies, it could be predicted that trait RS would affect response bias rather than detection sensitivity in judgment of cues of social rejection. In sum, our findings imply that evolutionary and RS theories emphasize different aspects of sensitivity to social rejection cues: evolutionary theory reflects detection capability whereas trait RS reflects vigilance and defensive responses to social rejection cues—and possibly bias in their interpretation.

Our findings extend previous RS research by showing that people with high trait RS evince heightened vigilance (i.e., increased LPP amplitude) and defensive responses (i.e., decreased P1 amplitude) to cues of social rejection at the neural level. Previous studies have used subjective and behavioral measures to provide a wealth of evidence about vigilance and responses to social rejection (e.g., Downey and Feldman, 1996; Downey et al., 1998b, 2004; Downey and Romero-Cayas, 2005; Berenson et al., 2009; Romero-Canyas et al., 2010). By using high temporal resolution electrophysiological methods, we showed that the defensive response occurs very fast (i.e., around 100 ms) and vigilance persists relatively longer (i.e., around 600 ms).

Our findings also provide novel insight on neurocognitive aspects of trait RS by showing that people with high RS have *decreased* P1 amplitude in response to disgusted faces. This finding is inconsistent with a recent study that reported people with high RS showed *increased* P1 amplitude in response to facial stimuli (Ehrlich et al., 2015). These seemingly contradictory results may be explained by at least two ways. First, the characteristics of our participants may have affected our results. Ehrlich et al. (2015) recruited female participants with high (top 20th percentile of enrolled database: *N* = 16, *M* = 13.56, *SD* = 2.41) or average (40–60th percentile: *N* = 14, *M* = 8.55, *SD* = 0.68) RS. Thus, it is possible that gender or grouping had an effect on our results for the P1. To test possible gender effects, we conducted a partial correlation analysis controlling sex, but the negative correlation between trait RS and P1 amplitude in response to disgusted face was still significant (*r* = -0.38, *p* = 0.025). To test possible a grouping effect, we divided participants into three groups on the basis of trait RS—low (*N* = 11, *M* = 10.01, *SD* = 1.63), medium (*N* = 12, *M* = 13.31, *SD* = 0.98), and high (*N* = 12, *M* = 17.14, *SD* = 1.69)—and conducted a 3 (RS: low vs. middle vs. high) × 3 (facial expression: neutral vs. smiling vs. disgusted) ANOVA on P1 amplitudes. We found no evidence that higher RS scores were associated with *increased* P1 amplitude. Thus, gender and grouping effects could not explain the observed inconsistency. It is also possible that stimuli or task differences may have made a difference between the previous and present findings. Ehrlich et al. (2015) used a modified dot-probe task and presented a neutral face with direct or averted eye gaze. In contrast, we used emotional faces: neutral, smiling, and disgusted faces. Thus, stimuli and task differences may have modulated the neurocognitive responses specific to trait RS. Some evidence supports this argument. For example, neural responses to emotional faces were modulated by task (Cohen Kadosh et al., 2010). In addition, trait RS scores were *negatively* correlated with amygdala responses to disgusted faces (Burklund et al., 2007); people with high RS showed less amygdala responses. Our findings conform to this study because the P1 amplitude is also modulated by the amygdala (e.g., Vuilleumier, 2005; Rotshtein et al., 2010). Thus, early neurocognitive responses specific to trait RS appear to be modulated by the stimuli and tasks used in the studies. Because little research has investigated the neurocognitive responses of trait RS using ERPs, future studies need to clarify the boundary at which people with high trait RS show *increased* or *decreased* P1 amplitudes in response to threatening cues.

In this study, we presented evidence of a disassociation between RS and RDC. However, it is possible that other dispositions, such as self-esteem and belongingness (e.g., Leary et al., 1995, 2013; DeWall et al., 2011; Beekman et al., 2015) might also be related to RDC. We believe that these two dispositions could affect the processing stages of social rejection, differently from RDC. RDC reflects individual differences in detecting social rejection cues, whereas self-esteem and belongingness might mainly reflect emotional and stress responses to social rejection cues (e.g., Onoda et al., 2010; Beekman et al., 2015). For example, people with high belongingness showed increased cortisol responses as a reaction to social exclusion (Beekman et al., 2015): moreover, people with high self-esteem showed increased self-reported social pain and dACC activity (Onoda et al., 2010). According to the intrapersonal and interpersonal process model of social exclusion (Kawamoto et al., 2015), social rejection cues cause three intrapersonal process stages—detection, appraisal, and regulation. On the basis of this model, we consider that RDC might reflect individual differences in the detection stage, whereas self-esteem and belongingness might influence the appraisal and regulation stages in response to social rejection cues. Empirical evidence regarding responses to social inclusion cues also clarifies differences between self-esteem/belongingness and RDC. We demonstrated that RDC is related to N170 amplitude only in response to social rejection cues, whereas previous studies have shown that self-esteem and belongingness are susceptible to social cues regardless of the valance, including social inclusion cues (e.g., Gardner et al., 2000; Somerville et al., 2010). However, given that the direct rejection subcomponent scores of RDC was related to N170 amplitude in response to smiling faces, it is possible that RDC subcomponents in part overlapped with belongingness and self-esteem. We thus believed that both self-esteem and belongingness are not be related, or if they were related, they would be only weakly related to RDC.

It should also be noted that, we unexpectedly found that RDC comprised three subcomponents. In addition, we also found that the direct rejection subcomponent was strongly related to evolutionary benefits and detection-related processes, such as increased perceived social inclusion experiences and enhanced N170 amplitude in response to disgusted faces, as compared to indirect rejection and minimal rejection subcomponents. We do emphasize that none of RDC subcomponents were related to RS, which again indicated that RS is not related to the ability to detect social rejection cues.

### Limitations and Future Directions

We acknowledge several limitations of the present study. First, although we found that RDC is related to perceive inclusion experiences, we did not find a significant relationship between RDC and depression. Thus, the precise nature of the relationship between RDC and mental health remains open to debate. Nevertheless, we emphasize that the observed relationship between RDC and perceived social inclusion experiences implies that the capability of detecting social rejection cues has some benefit in interpersonal relationships.

Second, we only focused on disgusted faces as social rejection cues. Recent studies have implied that facial expressions of anger and disapproval could also communicate social rejection (e.g., Burklund et al., 2007; Heerdink et al., 2015). In addition, trait RS produces different neural responses to different facial expressions: RS scores are positively correlated with dACC in response to a disapproval expression, whereas RS scores are negatively correlated with dACC and amygdala in response to an angry face (Burklund et al., 2007). Thus, future research should investigate whether our result is specific to the disgusted face by the use of other negative facial expressions, such as anger and disapproval.

Third, we did not directly examine social rejection experiences using experimental manipulations, such as the Cyberball task (Williams et al., 2000) or future life imagination (Twenge et al., 2001). Although we believe our study design was adequate to investigate the phenomena of interest, future research is required to investigate these in more detail, given that social rejection causes multiple neural responses and subsequent behaviors. For example, social rejection induces detection, appraisal, and regulation processes in our brain (e.g., Eisenberger et al., 2003; Yanagisawa et al., 2011a,b; Kawamoto et al., 2015). In addition, people can behave in both prosocial and antisocial ways following social rejection (e.g., Twenge et al., 2001; Maner et al., 2007; DeWall et al., 2009b; Chester et al., 2014). Thus, future studies could profitably investigate social rejection processing—detection, appraisal, and regulation processes—and subsequent behaviors, both prosocial and antisocial, following social rejection.

Fourth, the positive correlation between trait RS and LPP amplitude in response to smiling faces requires careful interpretation. Trait RS has been conceptualized as an aberrant response to social rejection cues, but not to social inclusion cues, which include smiling faces (e.g., Downey and Feldman, 1996; Romero-Canyas et al., 2010). Thus, our unexpected findings for smiling faces may not reflect the influence of trait RS *per se*. There are at least three possible explanations for this result. First, other aspects of personality, such as belongingness and self-esteem, may have been involved. Previous studies have indicated that both trait belongingness and self-esteem modulate responses to cues of social inclusion (e.g., Murray et al., 2001, 2002; Pickett et al., 2004), and these traits are significantly related to trait RS (e.g., Downey and Feldman, 1996; Pickett et al., 2004). Although trait RS has unique effects even after the other traits are controlled (Downey and Feldman, 1996; Romero-Canyas et al., 2010), we cannot rule out this possibility, as we only measured RS and RDC. Second, there may be some cultural differences in trait RS. For example, previous studies have revealed that trait RS scores are higher in Japan than in America (Garris et al., 2011; Sato et al., 2014). In addition, relational mobility partly explains the relationship between culture and RS scores (Sato et al., 2014). To the best of our knowledge, no study has found qualitative differences in trait RS among cultures; however, cultural difference may have had some effect on our results. For instance, cues of social inclusion may also important for people in Japan with high trait RS because relational mobility is low in Japan. Third, trait RS modulates the neural responses to cues of social inclusion. Previous studies have indicated that a history of social rejection can modulate the response to smiling faces (Vrticka et al., 2008; Cacioppo et al., 2009). Given trait RS is assumed to develop within a context of early social rejection experiences and a lack of adequate relationships with others (Feldman and Downey, 1994; Downey et al., 1997), trait RS may directly effect the processing of cues of social inclusion. In either case, future studies should investigate the relationship between trait RS and responses to cues of social inclusion in more detail by focusing on other cultures and personality variables.

Fifth, the sample size of Study 2 was relatively small. Therefore, it is possible that the weak, or null effects observed in Study 2 were caused by the small sample size. Also, RS and RDC were not significantly correlated in Study 2. This, however, could not have been caused by the small sample size, because the result of Study 1 that included a larger sample size (*N* = 116) also showed a non-significant relationship between RS and RDC. Furthermore, patterns of correlation between trait measures and N170 amplitudes in response to disgusted faces were similar. We do emphasize that partial correlation analysis clarified that N170 amplitude in response to disgusted faces was only related to RDC.

Sixth, RDC scales need further elaboration. Although we mainly focused on the total RDC score, confirmatory factor analysis showed that the one factor model did not fit well. We believe that our RDC scale is meaningful, because RDC total scores had high reliability (Cronbach α = 0.91, test–retest reliability: *r* = 0.72) and they were related to detection specific electrophysiological responses (i.e., N170 amplitude in response to disgusted face). Nevertheless, future studies using larger sample sizes should improve the RDC scale, because confirmatory factor analysis is highly influenced by the sample size (Breckler, 1990; La Du and Tanaka, 1995).

Finally, although emotional effects on ERPs were not the main focus of our study, such effects were not strong. It is suggested that future studies should be conducted to investigate if our results regarding ERP can be replicated by including a larger sample size and other social threat related stimuli, such as angry faces. We interpreted the results of LPP based on RS theory. Future research is needed to clarify emotional effects of LPP in more detail, because as indicated by Hajcak et al. (2010), LPP has partly overlapping spatial and temporal distributions with the other positive ERP component, P3 (P300).

## Conclusion

The present study revealed that trait RS influences vigilance and defensive responses to cues of social rejection, rather than the capability of detecting those cues. This capability in fact seems to be beneficial in interpersonal functioning by leading to greater perception of inclusion experiences. We believe that our findings have clear implications for both theory and practice. If the ability to detect social rejection cues has some benefit, research and clinical attention should focus on regulation of rejection-related anticipation and other exaggerated responses to rejection cues. Our findings strongly suggest the importance of investigating multiple aspects of sensitivity to social rejection: vigilance, detection capability, and defensive response.

## Conflict of Interest Statement

The authors declare that the research was conducted in the absence of any commercial or financial relationships that could be construed as a potential conflict of interest.
