# Supplementary material for: Trait rejection sensitivity is associated with vigilance and defensive response rather than detection of social rejection cues
Source: Front Psychol. 2015 Oct 2;6:1516. doi: 10.3389/fpsyg.2015.01516 (PMC4591508; doi:10.3389/fpsyg.2015.01516)
Supplement: Supplementary file 1 [file Data_Sheet_1.DOCX]

**Appendix**

Rejection Detection Capability (RDC) Scale

Each of the items below describes things people sometimes encounter in everyday life. People occasionally notice discrepancies in situations because the situation is unwanted, or relatively unusual. How much discrepancy would you notice in each of the thirteen situations below, if you were to encounter this situation? Please respond by choosing a number from 1 (*not at all*) to 9 (*very much*).

1. When you ask your friends to attend a class with you, they refuse (c).
2. When you extend greetings to friends, they ignore you (c).
3. Your friends all go to hang out somewhere but exclude you (a).
4. When you talk with your friends, they are less responsive to you than to others (a).
5. When you send an e-mail to your friends, you get no replies (b).
6. Your friends go for a meal without you (a).
7. You arrange to meet your friends, but they cancel or fail to show up (c).
8. Your friends go out drinking but leave you out (a).
9. Your friends leave you out of heated or animated conversations (b).
10. Your friends complain about you (c).
11. When your friends are talking, you are left out of the conversation (b).
12. You talk to your friends but they ignore you (c).
13. When you call a friend, he/she refuses to answer the phone (b).

Note: (a) indirect rejection, (b) minimal rejection, (c) direct rejection
